# Family aspects, physical fitness, and physical activity associated with mental-health indicators in adolescents

**DOI:** 10.1186/s12889-021-12403-2

**Published:** 2021-12-30

**Authors:** Lucía Lema-Gómez, Carlos Mario Arango-Paternina, Cleiber Eusse-López, Jorge Petro, Jose Petro-Petro, Milton López-Sánchez, Willinton Watts-Fernández, Fabio Perea-Velásquez

**Affiliations:** 1 Departamento de Cultura Física, Research Group in Physical Activity, Sports and Health Sciences (GICAFS), Universidad de Córdoba, Montería, Colombia; 2 Instituto Universitario de Educación Física, Universidad de Antioquia, Medellín, Colombia

**Keywords:** Anxiety, Health-related quality of life, Mental health, Physical activity, Physical fitness, Sedentary behavior, Stress

## Abstract

**Background:**

The objective of the study was to analyze the associations of family aspects, physical fitness, and physical activity with mental-health indicators in a sample of adolescents from Colombia.

**Methods:**

A cross-sectional study carried out in a sample of 988 adolescents (11-17 years-old) from public schools in Montería. Mental-health indicators were evaluated: Stress, depression, anxiety, happiness, health-related quality of life (HRQL), and subjective wellness. Family aspects included family affluence, functionality, and structure. These variables, along with physical activity and screen time, were measured with questionnaires. A fitness score was established by assessing the components of fitness: Flexibility, cardiorespiratory fitness, grip strength, and lower-limb strength. Associations were analyzed by multivariate linear regression models.

**Results:**

Nuclear family structure was associated with lower stress level (− 1.08, CI: − 1.98 - -0.18), and family functionality was associated with all the studied mental-health indicators (Stress: -0.11, CI: − 0.17 - -0.06; depression: -0.20, CI: − 0.25 - -0.16; trait anxiety: -0.13, CI: − 0.18 - -0.09; state anxiety: -0.12, CI: − 0.17 - -0.08; happiness: 0.09, CI: 0.07 - 0.1; HRQL: 1.13, CI: 0.99 - 1.27; subjective wellness: 1.67, CI: 1.39 - 1.95). Physical activity was associated (β, 95% Confidence Interval (CI)) with depression (− 0.27, − 0.57 - -0.02), trait anxiety (− 0.39, CI: − 0.65 - -0.13), state anxiety (− 0.30, CI: − 0.53 - -0.07), happiness (0.14, CI: 0.06 - 0.22), HRQL (3.63, CI: 2.86 – 4.43), and subjective wellness (5.29, CI: 3.75 – 6.83). Physical fitness was associated with stress (− 0.80, CI: − 1.17 - -0.43), state anxiety (− 0.45, CI: − 0.73 - -0.17), and HRQL (1.75, CI: 0.82 - 2.69); screen time was only associated with stress (0.06, CI: 0.02 - 0.11).

**Conclusions:**

Family aspects were associated with mental health indicators, especially family functionality which was associated all mental-health indicators. Similarly, fitness, physical activity, and screen time were associated with the studied indicators of mental health. Particularly, physical activity was associated with all the mental-health indicators, except stress, which was only associated with screen time. Physical fitness was associated with stress, anxiety, and HRQL. Future studies could explore the causal relationships of fitness, physical activity and screen time with mental health in adolescents.

## Background

Mental health is an invaluable resource for personal development and adequate levels of social functioning. Currently, mental-health problems represent a prominent field for public health. This is mainly due to its high prevalence globally and to its adverse effects on the burden of global morbidity [1] and mortality [2]. It has been estimated that one in three adults has experienced alterations in their mental health in the course of their life [3]. The study of mental health in the school-age population is relevant due to its role in the development, in all its aspects – physical, social, intellectual and emotional; also, half of these alterations begin during adolescence [4]. Unfortunately, mental well-being is an elusive condition for much of the adolescent population. For example, it has been estimated that between 10 and 20% of adolescents on the planet have poor mental health [5]. In the case of Colombia, this estimate is considerably higher, reaching 33% [4].

Historically, the study of mental health has centered its interest on psychopathological symptoms and negative mental health conditions such as depression, anxiety, stress among others. More recently, positive psychology has emerged and incorporated positive domains and components to mental health. Here, the constructs of well-being, subjective wellness, life satisfaction, happiness and the like, are the focus of attention [6]. These constructs may be understood as cognitive and affective processes around life satisfaction and positive emotional experiences [7]. These positive mental processes may protect against the onset of mental health disorders [8].

Mental health is a complex concept; therefore, its determinants and associated factors are multiple, among which stand out personal aspects (state of health, previous experiences, habits related to health), family (quality of the family environment, economic conditions), and social (quality of social relationships with peers and friends), the built environment, among others [9]. In particular, previous studies have documented that high levels of physical activity, low levels of screen time, and sufficient sleep time are behaviors associated with higher levels of mental health [10, 11]. Furthermore, similar associations of physical activity with depression [12] and happiness [13], and physical fitness with mental health [14] have been reported. In addition, randomized controlled trials have documented the positive effects of physical activity on depression [15] and self-esteem [16]. Regarding the family, it has been pointed out that the nuclear family structure generates a protective environment for mental health in young people [17] and that family environment with economic deprivation can negatively affect adolescent mental health [18].

These antecedents reflect an advance in the understanding of factors associated with mental health in adolescents. However, more research attention is needed in low- and middle-income countries. Consequently, the objective of this study was to explore the associations of family aspects, physical fitness, and physical activity with mental-health indicators in a sample of adolescents in Colombia. In this study, mental-health indicators are explored, including negative entities such as stress, depression and anxiety, and positive entities such as happiness, health-related quality of life (HRQL), and subjective well-being. Identifying these factors associated with mental-health indicators could inform early interventions aimed at strengthening adolescents’ mental health.

## Methods

In this study, information was used from the project “Determinants of academic achievement, health, and wellness in school-age children” (De-Redes, 2018), and conducted in the city of Montería, Colombia. It is an observational study with a cross-association design carried out in adolescents from public schools, between 11 and 17 years old. The city has 63 public schools, of which eight urban and two rural were randomly selected. In each school, a course per grade, between grades 7 and 10, was randomly selected. In each course, all students of that course were invited to participate. The sample consisted of 988 students who voluntarily participated in the study; they signed the informed assent and their parents authorized their participation by signing the informed consent. The study was conducted following the guidelines of Declaration of Helsinki and its protocol was approved by the Research Ethics Committee of the University of Antioquia’s University Institute of Physical Education.

## Measurements

### Mental-health indicators

Seven mental-health indicators were measured, taken as outcome variables: the level of stress was measured with the Spanish version of the Children’s Daily Stress Inventory (CDSI), consisting of 25 items (score total between 0 and 25) [19]. To estimate the level of depression, the abbreviated version of the inventory of depression in children (Children’s Depression Inventory, CDI:S) was used, made up of ten items (total score between 0 and 20) [20]. Anxiety was measured with the State-Trait Anxiety Inventory for Children (STAIC), consisting of six items for each type of anxiety (total score between 5 and 24) [21]. Happiness was evaluated with the Subjective Happiness Scale, composed of four items (total score between 4 and 28, which was then averaged) [22]. HRQL was measured with the KidScreen-27 instrument, made up of 27 items (total score between 27 and 100) [23];]; and subjective well-being was estimated with the Spanish adaptation of the Personal Well-being Index- School Children (PWI-SC) [24], and applied to the Latin American context [25], composed of 12 items (total score between 0 and 120). All these instruments had acceptable levels of internal consistency (α_stress_ = .70, α_depression_ = .77, α_trait anxiety_ = .72, α_state anxiety_ = .70, α_happiness_ = .60, α_HRQOL_ = .91, and α_subjective wellness_ = .92).

### Family aspects

Three aspects of the family were analyzed: As a proxy for socioeconomic position, the Family Affluence Scale (FAS) was used; it inquiries about four elements that reflect the material conditions of the family: one’s own room, Internet access at home, number of computers and cars [26]. The responses were added and gave a score between 0 and 8, to which tertiles were computed, indicating a better economic position as the tertile increases. The family structure was investigated to identify three types of structure: nuclear, single parent, and other. Family functionality was explored using the Family Apgar instrument [27], which contains five items exploring how satisfied the adolescent is with their family functions. Each item can be scored between zero and five, resulting in a score ranging from zero to 25.

### Physical fitness

Physical fitness was evaluated in different capacities. Flexibility was evaluated with the sit-and-reach test, which measures the flexibility of the lower back region and the hamstring muscles, and its values ​​were recorded in centimeters [28]. The evaluation of the explosive strength of the lower limbs was carried out with the long-jump test, performing a forward jump with the greater impulse of the lower limbs, and the distance reached in centimeters was recorded [29]. Cardiorespiratory fitness was measured with the 20-m shuttle-run test, designed to evaluate the ability of the cardiorespiratory system to transport and use oxygen [30]; it consists of going and coming back in a space of 20 m, at an initial speed of 8.5 ∙ km h-1, with increments of 0.5 km h-1 every minute until reaching maximum performance, recording the number of laps completed. With this information, the equation was applied to calculate the maximum oxygen consumption [31]. The grip strength was evaluated with the Jamar Handgrip Dynamometer (Sammons Preston, Bolingbrook, Illinois, USA.). The participant executed the test in each hand; the average was recorded, and the values ​​were normalized by body weight. Each of the four physical-fitness variables was standardized for sex and age, by calculating z-scores, which were averaged to obtain a fitness z-score.

### Physical activity and screen time

Physical activity was measured with the Spanish version of the Physical Activity Questionnaire for Adolescent (PAQ-A) [32], of which acceptable correlation values ​​have been reported with accelerometry [32]. In the questionnaire, a score between 1 and 5 was obtained; the higher the values, the higher the physical activity level. For screen time, the Sedentary Behavior Questionnaire (SBQ) [33], in which different domains of sedentary behavior were explored, including screen time made up of the use of television, computer, and video -games. The responses in these domains were added to obtain the number of hours of screen time per day. The instrument is valid for the classification of sedentary behavior [34].

### Sociodemographic variables

The body weight and height of the participants were measured to obtain the Body Mass Index (BMI); a Health or Meter scale with a precision of 200 g was used for body weight, and a Seca-brand height rod was used for height, measured in millimeters. Classification of BMI was based on z-scores from World Health Organization reference measurements [35]. The participants reported their age and sex, and the urban or rural location of the schools was recorded. Urban and rural areas are defined by the government based on political-administrative categories of the degree of urbanization. Urban areas are characterized by having a high degree of urbanization and high residential density. While rural areas are defined as the residual of the urban, that is, areas with scattered residences and low residential density [36].

## Analysis

Descriptive statistics, proportions, and mean ± standard deviation were calculated after verifying the normal distribution in the variables considering the values ​​of asymmetry and kurtosis (values ​​between − 2 and + 2). Mean differences of mental health scores according to categorical variables were tested with independent samples T-Tests and one-way ANOVA and post hoc Tukey tests. For the analysis of the association between the variables analyzed and the mental-health indicators, the bivariate associations (raw) were initially explored, and then the multivariate linear regression models (adjusted) were constructed. Bivariate and multivariate associations were performed separately for each mental-health indicator. This is, binary associations between each one of the independent variables (sociodemographic variables, family aspects, physical fitness, physical activity, and screen time) and the seven mental health indicators were analyzed. Then, for the multivariate models and following the Homer-Lemeshow criterion [37], variables with *p*-values ​​ < .25 in the bivariate association were selected and included in the models. For the variable of family structure, which have three categories, dummy variables were created to fit the linear models. In total, seven separate models were analyzed, one for each mental health indicator. Interaction term between sex and age were explored and its associations with all of the analyzed mental health indicators (a three-way interaction) were not significant. The 95% confidence intervals were obtained, and the significance level was established at *p*-value <.05. The analyzes were carried out with the SPSS Statistical Program for Windows v.24 (IBM Corp., Armonk, N.Y., USA).

## Results

The descriptive characteristics are shown in Table 1. In total, 988 adolescents with an average age of 14.9 ± 1.5 years participated; girls made up 53.1% of the sample (*n* = 525), and about four out of every five adolescents were students from schools located in the urban zone. The FAS tertiles with the highest proportion of adolescents were – in their order – the second (41.6%), the third (31.6%) and the first (26.8%). One in three adolescents reported belonging to a nuclear family, and 26.1% (*n* = 258) reported belonging to a single-parent family, and the rest (7.5%) (*n* = 74) to another type of family structure. A prevalence of overweight of 16.3% (*n* = 161) was found. Table 1 also shows the results of the scores obtained in the measurement scales of the mental-health indicators, physical fitness, and physical activity level, as well as the average number of hours per day of screen time (7.2 ± 4.3).

**Table 1:** Sample characteristics

Variables	*n*		%	
Sex				
Boys	463		46.9	
Girls	525		53.1	
Location				
Rural	169		17.1	
Urban	819		82.9	
Family Affluence Scale (tertiles)				
First	265		26.8	
Second	411		41.6	
Third	312		31.6	
Family structure				
Other	74		7.5	
Single parent	258		26.1	
Nuclear	656		66.4	
Overweight				
No	827		83.7	
Yes	161		16.3	
	*Mean (SD)*	*Min. - max.*	*Asymmetry (SE = .08)*	*Kurtosis (SE = .16)*
Age	14.9 (1.5)	11 - 17	.18	−.46
Apgar score	13.9 (4.2)	0 - 25	−.58	−.12
Stress score	8.4 (3.9)	0 - 22	.38	.01
Depression score	3.2 (3.2)	0 - 20	1.27	1.96
Trait-anxiety score	11.2 (3.2)	6 - 24	.51	.12
State-anxiety score	13.0 (2.9)	6 - 23	.08	−.10
Happiness score	4.8 (1.1)	1 - 7	−.29	.11
HRQL score	74.0 (11.8)	35.3 - 100	−.41	−.10
Subjective-wellness score	94.2 (20.7)	0 - 120	−1.1	1.40
Physical Fitness (z-score)	0.0 (0.6)	−2.0 - 2.8	−.04	.18
Physical-activity score	2.5 (0.8)	1.0 - 5.0	.10	−.25
Screen time (hours per day)	7.2 (4.3)	0 - 17	.23	−1.00

HRQL: Health-related quality of life

SD: Standard deviation

SE: Standard error

Mean differences of mental-health indicators according to categories of independent variables are shown in Table 2. Compared to boys, girls reported higher levels of stress, depression, trait anxiety, and state anxiety. Boys had higher scores of HRQL and subjective wellness. Students from urban schools reported higher scores of stress and depression, and lower subjective wellness score than students from rural schools. Compared to adolescents from nuclear families, adolescents with a family structure categorized as other, reported higher levels of stress (pairwise comparisons). There were differences in levels of HRQL across categories of family structure, but pairwise comparisons were not statistically significant.

**Table 2:** Mean differences of mental-health indicators according to categories of independent variables

	Stress	Depression	Trait anxiety	State anxiety	Happiness	HRQL	Subjective wellness
Variables	Mean (SD)	Mean (SD)	Mean (SD)	Mean (SD)	Mean (SD)	Mean (SD)	Mean (SD)
Sex							
Boys	7.8 (3.8)*^a^	2.6 (2.8)*^a^	10.7 (3.0)*^a^	12.2 (2.7)*^a^	4.8 (1.0)	77.4 (10.9)*^a^	97.0 (17.8)*^a^
Girls	8.9 (3.9)	3.8 (3.3)	11.6 (3.3)	13.7 (2.9)	4.7 (1.2)	71.0 (11.7)	92.0 (22.8)
Location							
Rural	7.2 (3.7)*^a^	2.1 (2.5)*^a^	11.1 (2.9)	12.8 (2.6)	4.8 (1.0)	75.3 (11.9)	98.0 (21.6)*^a^
Urban	8.7 (3.9)	3.4 (3.2)	11.2 (3.3)	13.0 (3.0)	4.8 (1.1)	73.7 (11.7)	93.5 (20.5)
Family Affluence Scale (tertiles)							
First	8.5 (3.9)	3.0 (3.1)	11.1 (2.9)	13.1 (2.9)	4.8 (1.1)	73.2 (12.0)	94.4 (20.1)
Second	8.5 (4.0)	3.2 (3.1)	11.1 (3.2)	13.0 (2.9)	4.8 (1.1)	73.6 (11.5)	93.2 (21.96)
Third	8.2 (3.7)	3.4 (3.3)	11.3 (3.5)	12.9 (3.0)	4.7 (1.1)	75.2 (11.9)	95.4 (19.0)
Family structure							
Other	9.7 (3.6)*^b^	3.8 (3.4)	11.7 (4.0)	13.5 (2.8)	4.6 (1.1)	71.4 (12.8)*^b^	94.0 (25.5)
Single parent	8.8 (4.0)	3.0 (3.0)	11.3 (3.3)	13.0 (3.0)	4.8 (1.1)	73.3 (11.7)	93.0 (21.2)
Nuclear	8.1 (3.8)	3.2 (3.2)	11.1 (3.1)	13.0 (2.9)	4.8 (1.1)	74.6 (11.6)	94.7 (20.0)
Overweight							
No	8.4 (3.9)	3.2 (3.2)	11.2 (3.2)	12.9 (2.9)	4.8 (1.1)	74.1 (11.7)	94.8 (20.1)
Yes	8.7 (3.8)	3.4 (3.2)	11.2 (3.4)	13.4 (3.1)	4.8 (1.2)	73.5 (12.3)	91.3 (23.4)

HRQL: Health-related quality of life. * Differences statistically significant

^a^ Mean differences tested with independent samples T-Tests. ^b^ Mean differences tested with one-way ANOVA

The results of the bivariate (crude) linear associations between the variables studied and the mental-health indicators are shown in Table 2. The coefficients indicate that as age was positively associated with stress, trait and state anxiety, and negatively associated with HRQL and subjective well-being. Similarly, being a girl was significantly associated with higher stress levels, depression, trait and state anxiety, and lower levels of HRQL and subjective well-being. Belonging to urban schools was significantly associated with higher levels of stress and depression and lower levels of subjective well-being. The occurrence of overweight was significantly associated with lower subjective well-being values.

Regarding family aspects, shown in Table 3, it was found that FAS tertiles were positively associated with HRQL. Belonging to a single parent family was negatively associated with depression, and being member of a nuclear family was negatively associated with stress and positively associated with HRQL. Family’s functionality score was negatively associated with stress, depression, trait and state anxiety, and positively associated with happiness, HRQL, and subjective well-being. Physical fitness was negatively associated with stress and state anxiety and positively associated with HRQL. Regarding physical activity, it was found that this behavior was negatively associated with stress, depression, trait and state anxiety and positively associated with happiness, HRQL, and subjective well-being. Finally, screen time was positively associated with stress and anxiety.

**Table 3:** Bivariate linear associations between studied variables and mental-health indicators

	Stress	Depression	Trait anxiety	State anxiety	Happiness	HRQL	Subjective wellness
Variables	β (95% CI)	β (95% CI)	β (95% CI)	β (95% CI)	β (95% CI)	β (95% CI)	β (95% CI)
Age	0.46 (0.3; 0.6)**	0.08 (− 0.1; 0.2)	0.26 (0.1; 0.4)**	0.14 (0.01; 0.26)*	−0.03 (− 0.1; 0.0)	−1.41 (−2.0; − 0.9)**	−1.80 (−2.7; − 0.9)**
Sex (Ref. Boys)	1.14 (0.7; 1.6)**	1.24 (0.8; 1.6)**	0.82 (0.4; 1.2)**	1.47 (1.11; 1.82)**	− 0.06 (− 0.2; 0.1)	−6.44 (− 7.8; − 5.0)**	−5.24 (− 7.8; − 2.7)**
Location (Ref. Rural)	1.48 (0.8; 2.1)**	1.37 (0.8; 1.9)**	0.07 (− 0.5; 0.6)	0.27 (− 0.22; 0.75)	− 0.05 (− 0.2; 0.1)	−1.61 (− 3.6; 0.3)	− 4.41 (− 7.8; − 1.0)*
Overweight (Ref. Normal)	0.30 (− 0.4; 1.0)	0.24 (− 0.3; 0.8)	0.05 (− 0.5; 0.6)	0.48 (− 0.01; 0.98)	0.07 (− 0.1; 0.2)	− 0.53 (− 2.5; 1.5)	−3.51 (− 7.0; − 0.0)*
Family Affluence Scale (tertiles)	−0.12 (− 0.4; 0.2)	0.22 (− 0.0; 0.5)	0.08 (− 0.2; 0.3)	−0.10 (− 0.34; 0.13)	−0.03 (− 0.1; 0.1)	0.99 (0.0; 1.9)*	0.57 (− 1.1; 2.3)
Family structure (Ref. Other)							
Single parent	−0.98 (− 2.0; 0.0)	−0.83 (− 1.6; − 0.0)*	−0.35 (− 1.2; 0.5)	−0.57 (− 1.3; 0.2)	0.21 (− 0.7; 0.5)	1.95 (− 1.1; 5.0)	− 1.02 (− 6.4; 4.3)
Nuclear	−1.61 (− 2.5; − 0.7)**	− 0.57 (− 1.3; 0.2)	− 0.58 (− 1.4; 0.2)	− 0.57 (− 1.3; 0.1)	0.11 (− 0.1; 0.4)	3.20 (0.4; 6.0)*	0.66 (−4.3; 5.6)
Family Apgar score	−0.13 (− 0.2; − 0.1)**	−0.22 (− 0.3; − 0.2)**	−0.15 (− 0.2; − 0.1)**	−0.14 (− 0.2; − 0.1)**	0.09 (0.1; 0.1)*	1.26 (1.1; 1.4)**	1.83 (1.5; 2.1)**
Physical Fitness (z-score)	−0.74 (− 1.1; − 0.4)**	−0.25 (− 0.6; 0.1)	−0.11 (− 0.4; 0.2)	−0.48 (− 0.8; − 0.2)**	0.06 (− 0.0; 0.2)	1.94 (0.8; 3.1)**	1.41 (− 0.6; 3.4)
Physical activity score	−0.43 (− 0.7; − 0.1)**	−0.61 (− 0.9; − 0.4)**	−0.63 (− 0.9; − 0.4)**	−0.70 (− 0.9; − 0.5)**	0.20 (0.11; 0.3)**	5.56 (4.7; 6.4)**	7.11 (5.6; 8.6)**
Screen time (hours per day)	0.09 (0.0; 0.1)**	0.06 (0.0; 0.1)**	−0.02 (− 0.1; 0.0)	0.03 (− 0.0; 0.1)	0.00 (− 0.0; 0.0)	−0.05 (− 0.2; 0.1)	−0.09 (− 0.4; 0.2)

* Association statistically significant at *p* < 0.05

** Association statistically significant at *p* < 0.01

The results of the analysis of the multivariate linear associations (adjusted) between the variables studied and the mental-health indicators are shown in Table 4. The regression models included adjustment variables that had *p*-values ​​ < .25 in the bivariate association. After including the adjustment variables, it was found that age and screen time were positively associated with stress, and family’s functionality score, physical fitness, and physical activity were negatively associated with stress. Being a girl and belonging to urban schools were associated with higher stress score, and being a member of a nuclear family was negatively associated with lower stress score. The linear model for depression, shows that being a girl and belonging to an urban school were associated with higher depression level. Family’s functionality and physical activity scores were negatively associated with depression. The linear models for trait and state anxiety showed similar results. Both anxiety dimensions were positively associated with age, and negatively associated with family’s functionality and physical activity scores. Being a girl was associated with higher trait and state anxiety level. In addition, physical fitness was negatively associated with state anxiety score.

**Table 4:** Multivariate linear associations between studied variables and mental-health indicators

	Stress	Depression	Trait anxiety	State anxiety	Happiness	HRQL	Subjective wellness
Variables	β (95% CI)	β (95% CI)	β (95% CI)	β (95% CI)	β (95% CI)	β (95% CI)	β (95% CI)
Age	0.50 (0.3; 0.7)**	Not included	0.23 (0.1; 0.4)**	0.14 (0.0; 0.3)*	Not included	−1.2 (− 1.6; − 0.8)**	−1.56 (− 2.4; − 0.7)**
Sex (Ref. Boys)	1.09 (0.6; 1.6)**	0.95 (0.6; 1.3)**	0.58 (0.2; 1.0)**	1.23 (0.9; 1.6)**	Not included	−4.01 (−5.2; −2.8)**	−1.60 (− 4.04; 0.8)
Location (Ref. Rural)	1.41 (0.8; 2.0)**	1.18 (0.7; 1.7)**	Not included	Not included	Not included	−1.74 (− 3.4; − 0.1)*	− 3.98 (−7.1; − 0.9)*
Overweight (Ref. Normal)	Not included	Not included	Not included	0.22 (−0.3; 0.7)	Not included	Not included	−3.05 (−6.3; 0.2)
Family Affluence Scale (tertiles)	Not included	0.07 (−0.2; 0.3)	Not included	Not included	Not included	0.78 (−0.0; 1.6)	Not included
Family structure (Ref. Other)							
Single parent	−0.68 (−1.6; 0.3)	−0.34 (− 1.1; 0.4)	Not included	−0.25 (− 1.0; 0.5)	Not included	−0.39 (−2.8; 2.0)	Not included
Nuclear	−1.08 (−2.0; − 0.2)*	−0.1 (− 0.7; 0.7)	Not included	−0.22 (− 0.9; 0.4)	Not included	0.49 (− 1.8; 2.8)	Not included
Family Apgar score	−0.11 (− 0.2; − 0.1)**	−0.20 (− 0.2; − 0.2)**	−0.13 (− 0.2; − 0.1)**	−0.12 (− 0.2; − 0.1)**	0.09 (0.1; 0.1)**	1.13 (0.1; 1.3)**	1.67 (1.4; 1.9)**
Physical Fitness (z-score)	−0.80 (− 1.2; − 0.4)**	−0.27 (− 0.6; 0.0)	Not included	−0.45 (− 0.7; − 0.2)**	Not included	1.75 (0.8; 2.7)**	0.74 (− 1.1; 2.6)
Physical activity score	0.07 (− 0.2; 0.4)	−0.27 (− 0.5; − 0.0)*	−0.39 (− 0.6; − 0.1)**	−0.30 (− 0.5; − 0.1)*	0.14 (0.1; 0.2)**	3.65 (2.9; 4.4)**	5.29 (3.7; 6.8)**
Screen time (hours per day)	0.06 (0.0; 0.1)*	0.03 (−0.0; 0.1)	Not included	0.01 (−0.0; 0.0)	Not included	Not included	Not included

* Association statistically significant at p < 0.05

** Association statistically significant at p < 0.01

Regarding positive components of mental health, linear regression models showed that family’s functionality and physical activity scores were positively associated with happiness. Age was negatively associated with HRQL, and being a girl and belonging to urban schools were associated with lower HRQL score. Family’s functionality score, physical fitness, and physical activity were positively associated with HRQL. Finally, age was negatively associated with subjective wellness. Being a girl and belonging to urban schools were associated with lower subjective wellness score. Family’s functionality score and physical activity were positively associated with subjective wellness. BMI was not associated with any of the mental-health indicators analyzed.

## Discussion

The study’s objective was to analyze the associations of family aspects, physical fitness, and physical activity with mental-health indicators in a sample of adolescents from Colombia. These results indicated that adolescents’ mental health is strongly related with the characteristics of the family, with the levels of physical fitness, and with physical activity and sedentary behavior, aspects that will be discussed separately.

### Demographic factors, family factors, and mental-health indicators

The findings reveal that female sex was associated with higher stress, depression, trait and state anxiety scores, and with lower HRQL score. Similar results were reported in a systematic review indicating that girls presented more mental health problems than boys [38]. In addition, results showed that family affluence was associated with HRQL, that family structure was associated with stress, and that family functionality was associated with the seven mental-health indicators analyzed. These findings are consistent with previous studies. For example, previous studies have documented that a family with appropriate functionality exerts a protective effect against depression in schoolchildren [39] and that adolescents from families with poor functionality are more likely to report depressive symptoms [40].

Among the mechanisms postulated to understand the relationship between family functioning and mental health, it has been pointed out that families with hostile and neglectful relationships trigger alterations in the nervous and hormonal systems of their children, causing emotions of detachment, rejection, and alienation in them, negatively affecting their mental health [41]. On the other hand, a positive, supportive and cohesive family environment promotes optimal levels of mental health in adolescents [42].

### Fitness, physical activity, screen time, and mental-health indicators

In turn, physical fitness was associated with stress, state anxiety, and HRQL; physical activity was associated with all the mental-health indicators studied except stress, which was the only mental-health indicator found to be associated with screen time. The results of this study were consistent with previous reports regarding the association between physical fitness and mental health [14, 43]. Similar to the results of this study, associations have been indicated between physical activity with depression [12, 44], anxiety [44], happiness [13], with HRQL [45, 46], and with subjective well-being [47]. However, the absence of association of physical activity with anxiety and stress has been documented [12]. In addition, it has been reported that the association between physical activity and psychosocial problems are significantly stronger in boys than in girls [43]. In this regard, we found no evidence that the relationships between fitness, physical activity and mental health varied with sex. Associations of time spent watching television with depression [48, 49], anxiety [48], HRQL [50], and subjective well-being [51] have been documented in the literature, but these associations were not found in this study. Similar to that reported in the literature [12, 14], no associations were found between the BMI and mental-health indicators analyzed in this study.

As for the mechanisms that help explain the relationship between physical fitness and mental health, a biological mechanism has been suggested, indicating that aerobic exercise causes an antidepressant effect via the endocannabinoid system [52]. In turn, it has been suggested that physical activity causes an increase in the brain-derived neurotrophic factor (BDNF), and this increase is associated with a reduction in levels of anxiety and depression [53]. Additionally, a psychosocial mechanism has been postulated, taking into account that participation in sports is popular during adolescence [54], and this may facilitate the development of socialization skills, considered a protective factor for mental health [55]. Although the explanatory mechanisms for the relationship between sedentary behavior and mental health are not well established, a psychosocial mechanism has been suggested by pointing out that excessive screen time can induce feelings of loneliness when performed in settings of social isolation, thus leading to poor mental health [56]. A behavioral mechanism is also proposed in the literature, indicating that – as more time is spent on the screen –physical activities [56] and adequate sleep [57] are reduced, negatively affecting adolescent mental health.

## Implications of the findings

The findings reported in this study have implications on the intervention with physical activity and the mental health of adolescents in the family, school, and community contexts since – consistent with other studies – we have found an association between the level of physical fitness, physical activity, and mental health indicators, as well as the relationship between screen time and a relevant mental-health indicator for this population (i.e., stress). These associations may be further explored to evaluate the effects of interventions on mental health in adolescent population. In this sense, we propose that emphasis should be placed on strengthening, in quality and quantity, physical activity programs inside and outside of school, in order to promote physical activity habits, stimulate the development of health-related physical fitness and, as far as possible, reduce the time spent on the screen. At the same time, wellness programs in schools should consolidate, both individually and collectively, the integration of family aspects and the students’ lifestyles as a human-development strategy. On the other hand, these results establish the need to look for a cause-effect relationship between physical activity/physical fitness and variables of mental-health outcomes. In this sense, trials that contemplate causal-mediation analysis [58, 59], aimed at exploring variables that have a mediating role on the effect of physical exercise on mental health in young people, may contribute to the construction of robust evidence for researchers and health and exercise professionals [60].

## Limitations and strengths of the study

The results presented should be analyzed under the limitations of this study. In the first instance, it is highlighted that it is a cross-sectional study; therefore, any causal inference, for example, between physical fitness/physical activity with mental health, should be excluded. Second, health indicators, physical activity and screen time were evaluated through self-reporting questionnaires that, although validated, may present risks of information and classification bias. In the same way, it should be considered that this study does not record history or prevalence of any type of disease (physical or mental), economic situation, violence, or factors related to the family or community environment where the adolescent lives that, in a certain way, can have a confounding effect on the association presented here. Finally, school location (urban and rural) is a school-level variable that could be affecting mental health of students from the same school. With this, the assumption of independent observations may be compromised. The exploration of this aspect requires a multilevel analysis. However, this approach is beyond the scope of the article. On the other hand, this study presents methodological strengths, such as the size of the sample types implemented that allowed including adolescents from the urban and rural sectors, and that represents a predominantly low-income population, characteristic of a low-high-income country such as Colombia. In addition, various indicators of mental health were taken into account (i.e., level of stress, level of depression, anxiety, subjective happiness, HRQL, and subjective wellness), and simultaneously the level of physical activity, screen time (sedentary behavior), and health-related physical fitness were evaluated (i.e., grip strength and lower limbs, cardiorespiratory capacity, and flexibility), which allowed a more complete analysis to be presented.

## Conclusion

This study shows that family affluence was associated with HRQL, that family structure was associated with stress, and that family functionality was associated with all mental-health indicators. The level of physical activity was associated with all indicators of mental health, except stress. On the other hand, physical fitness was related to stress, anxiety, and HRQL, while screen time was only associated with stress. In line with other research, the study findings have important practical implications that reveal the importance of promoting mental health in adolescents through school and community programs that promote the practice of physical activity, the development of health-related physical fitness, and decreased screen time. On the other hand, more experimental evidence of the effect of physical exercise on mental-health indicators in adolescents is required, suggesting causal-mediation analysis to explain the relationship between physical activity and mental health, as well as the possible mechanisms involved.

## Data Availability

All data is available from the corresponding author on reasonable request.

## Abbreviations

BMI: Body mass index
CDI:S: Children’s Depression Inventory
CDSI: Children’s Daily Stress Inventory
FAS: Family Affluence Scale
HRQL: Health-related quality of life
PAQ-A: Physical Activity Questionnaire for Adolescent
PA: Physical activity
PF: Physical fitness
PWI-SC: Personal Well-being Index- School Children
SBQ: Sedentary Behavior Questionnaire
STAIC: State-Trait Anxiety Inventory for Children

## Declarations

### Relevant guidelines

The study was conducted following the guidelines of Declaration of Helsinki.
